# National Early Warning Score 2 (NEWS2) better predicts critical Coronavirus Disease 2019 (COVID-19) illness than COVID-GRAM, a multi-centre study

**DOI:** 10.1007/s15010-021-01620-x

**Published:** 2021-05-10

**Authors:** Giuseppe Vittorio De Socio, Anna Gidari, Francesco Sicari, Michele Palumbo, Daniela Francisci

**Affiliations:** 1 Department of Medicine and Surgery, Clinic of Infectious Diseases, “Santa Maria della Misericordia” Hospital, Università degli Studi di Perugia, Piazzale Lucio Severi 1, 06132 Perugia, Italy; 2 Department of Medicine, Clinic of Infectious Diseases, “Santa Maria” Hospital, 05100 Terni, Italy

**Keywords:** COVID-19, SARS-CoV-2, NEWS2, National Early Warning Score 2, COVID-GRAM

## Abstract

**Purpose:**

Clinical scores to rapidly assess the severity illness of Coronavirus Disease 2019 (COVID-19) could be considered of help for clinicians. Recently, a specific score (named COVID-GRAM) for severe acute respiratory syndrome-coronavirus-2 (SARS-CoV-2) infection, based on a nationwide Chinese cohort, has been proposed. We routinely applied the National Early Warning Score 2 (NEWS2) to predict critical COVID-19. Aim of this study is to compare NEWS2 and COVID-GRAM score.

**Methods:**

We retrospectively analysed data of 121 COVID-19 patients admitted in two Clinics of Infectious Diseases in the Umbria region, Italy. The primary outcome was critical COVID-19 illness defined as admission to the intensive care unit, invasive ventilation, or death. Accuracy of the scores was evaluated with the area under the receiver-operating characteristic curve (AUROC). Differences between scores were confirmed used Hanley–McNeil test.

**Results:**

The NEWS2 AUROC curve measured 0.87 (standard error, SE 0.03; 95% CI 0.80–0.93; *p* < 0.0001). The COVID-GRAM score AUROC curve measured 0.77 (SE 0.04; 95% CI 0.68–0.85; *p* < 0.0001). Hanley–McNeil test showed that NEWS2 better predicted severe COVID-19 (Z = 2.03).

**Conclusions:**

The NEWS2 showed superior accuracy to COVID-GRAM score for prediction of critical COVID-19 illness.

**Supplementary Information:**

The online version contains supplementary material available at 10.1007/s15010-021-01620-x.

## Introduction

A new Coronavirus, subsequently named severe acute respiratory syndrome-coronavirus-2 (SARS-CoV-2) widely spread on Wuhan City, Hubei province, since December 2019, causing a diffusive type of interstitial pneumonia. In the following months, it diffused all over the world, becoming a global issue and forcing the World Health Organization to declare Coronavirus Disease 2019 (COVID-19) as a pandemic in March 2020.

An early assessment of illness severity for stratification of patients with SARS-CoV-2 infection is a relevant clinical need. Recently, Liang et al*.* [1] developed an interesting, specific score risk for COVID-19 that may help predict critical illness (COVID-GRAM). Briefly, authors, analysing a wide Chinese nationwide cohort, through a least absolute shrinkage and selection operator (LASSO) and logistic regression, constructed a predictive risk score (COVID-GRAM).

During this emergency period, in our clinical practice, we evaluated the predictive value of national early warning score 2 (NEWS2) to identify high-risk patients for intensive care unit (ICU) admission [2]. The NEWS2 was primarily validated for death and intensive care need in septic patients. It is a modified version of NEWS, which is commonly used in British hospitals for identification of patients with a high risk of deterioration [3].

This study aimed to compare the diagnostic accuracy of COVID-GRAM score proposed by Liang et al*.* and NEWS2 for predicting critical COVID-19 illness in a cohort of patients hospitalised for SARS-CoV-2 in Umbria region.

## Methods

This study is a retrospective, multi-centre accuracy study. Consecutive adult COVID-19 patients admitted to the Clinic of Infectious Diseases of Perugia Hospital, Umbria region, Italy, and Clinic of Infectious Diseases of Terni Hospital, Umbria region, Italy, from March 01, 2020 to June 15, 2020, were recruited. All subjects provided informed oral consent to clinical data collection. Data were collected using the same database in both centres to guarantee the homogeneity of the cohort.

All adult patients (age ≥ 18 years old) with an established diagnosis of SARS-CoV-2 infection were included. [4] The infection was established for patients with at least a sample (nasopharyngeal swab, bronchoalveolar lavage, and sputum) positive for the SARS-CoV-2 (molecular diagnosis with real-time polymerase chain reaction, PCR). The study was approved by the Ethics Committee of the Umbria Region (protocol number 18344/20/OV).

Data on demographics, comorbidities and clinical presentation were obtained for each patient. COVID-GRAM and NEWS2 at hospital admission were calculated using medical records.

Exclusion criteria were missing information that did not permit to calculate NEWS2 or COVID-GRAM. The primary outcome was critical COVID-19 illness as defined by Liang et al*.* [1]: admission to the ICU, invasive ventilation, or death.

Data were summarised as mean with the respective standard deviation (SD) or median with the respective interquartile range (IQR).

Differences between groups were determined using the Mann–Whitney test, the Student’s *t* test, or the *Χ*^2^ as appropriate.

NEWS2 is based on aggregate scoring of the following parameters: respiratory rate, hypercapnic respiratory failure, supplemental oxygen, body temperature, systolic blood pressure, pulse rate and level of consciousness. The combination of these values provides a score between 0 and 20 [3].

COVID-GRAM is a composite score calculated using the following formula: COVID-GRAM risk score = (X-ray abnormality × 27.1464) + (age × 0.6139) + (hemoptysis × 33.6210) + (dyspnoea × 14.0569) + (unconsciousness × 34.4617) + (number of comorbidities × 10.3826) + (cancer history × 31.2211) + (neutrophil/lymphocyte ratio, N/L × 1.25) + (lactate dehydrogenase, LDH × 0.0534) + (direct bilirubin × 3.0605). Age, number of comorbidities, N/L, LDH and direct bilirubin are continuous variables, and the others are categorial variables (expressed with 1 if positive and 0 if negative) [1].

Performances of NEWS2 and COVID-GRAM were evaluated by receiver-operating characteristic (ROC) curve analysis, describing areas under curves (AUROC) with their 95% confidence interval (CI) and comparing them to the null hypothesis (area = 0.5) [5–7]. The probability value (*p*) of the ROC curve was assessed using the Mann–Whitney *U* test. The Hanley–McNeil test was performed to establish if differences between ROC curves area were significant: areas under the ROC curve with critical *Z* ratio ≥ 1.96 were considered different [7].

For each score, the optimal threshold was chosen basing on the Youden index [8]. In addition, the performance of the score at the optimal threshold was assessed by the corresponding sensitivity and specificity, positive and negative likelihood ratio, positive and negative predictive value, and accuracy. Each value is reported with 95% confidence intervals (CI).

In clinical practice, as defined in previous studies, two thresholds (5–7 and 56.6–138.4 for NEWS2 and COVID-GRAM, respectively) could better identify patients with low, medium and high risk of progression to critical COVID-19 [1, 2]. Indeed, the same values described above were calculated for the optimal clinical thresholds defined in the literature.

Statistical analyses were performed using Prism Graphpad 8 software. A *p* value < 0.05 was considered significant.

## Results

**Fig. 1:**
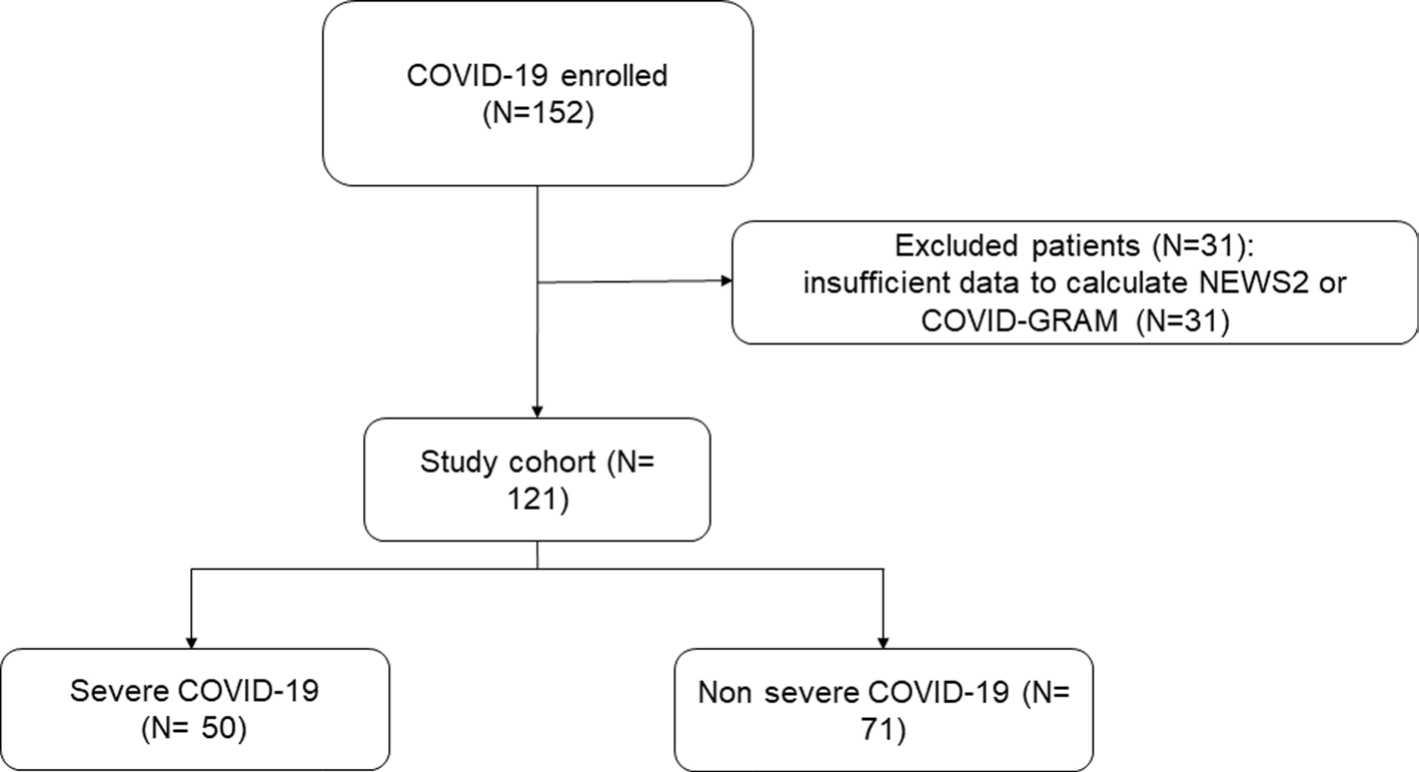
Patient selection flow chart

During the study period (as shown in Fig. 1), 152 patients were hospitalised for SARS-CoV-2 in Clinics of Infectious Diseases of Perugia and Terni. All the included patients were diagnosed with COVID-19 for the first time. Thirty-one were excluded due to insufficient data to calculate one or both scores at hospital admission. Therefore, 121 patients were included in the analysis. Among these, 50 (41.3%) patients presented critical illness. Analysing the subgroup of excluded patients, we found that 11/31 (35.5%) developed critical COVID-19, and among these, 6 patients died. Indeed, including these patients, the prevalence of severe COVID-19 would remain high (40.1%).

The two scores were calculated using data obtained at hospital admission. This timing corresponds to a median of 8 days (interquartile range 5–11 days) after symptoms onset.

Baseline characteristics of patients are summarised in Table 1. The mean age was 64.5, standard deviation (SD) ± 13.4 years; critical patients had a mean age slightly higher than non-critical patients (67.4, SD 10.7 years versus 62.5, SD 14.8 years, *p* = 0.04). Percentage of male is higher in critical patients compared to non-critical ones (78% and 56%, respectively, *p* = 0.01).

**Table 1:** Demographics, comorbidities and clinical presentation

	Total population	Critical illness	
		Yes	No
No. (%)	121	50 (41.3)	71 (58.7)
Age, mean (SD) [range], years	64.5 (13.4) [31–90]	67.4 (10.7) [48–89]	62.5 (14.8) [31–90]
Sex			
Male, no. (%)	79/121 (65.3)	39/50 (78.0)	40/71 (56.0)
Comorbidities, no. (%)			
0	59/121 (48.8)	23/50 (46.0)	36/71 (50.7)
1	39/121 (32.2)	15/50 (30.0)	24/71 (33.8)
2	14/121 (11.6)	8/50 (16.0)	6/71 (8.5)
3	8/121 (6.6)	4/50 (8.0)	4/71 (5.6)
4	1/121 (0.8)	0/50 (0)	1/71 (1.4)
5	0/121 (0)	0/50 (0)	0/71 (0)
Malignant disease, no. (%)	10/121 (8.3)	6/50 (12.0)	4/71 (5.6)
Hemoptysis, no. (%)	0/121 (0)	0/121 (0)	0/71 (0)
Dyspnoea, no. (%)	66/121 (54.5)	36/50 (72.0)	30/71 (42.3)
Unconsciousness, no. (%)	0/121 (0)	0/100 (0)	0/71 (0)
Neutrophil–lymphocyte ratio, median [IQR]	4.4 [2.7–8.2]	7.6 [4.1–12.1]	3.8 [2.1–5.3]
Lactate dehydrogenase, median [IQR], U/L	265.0 [190.0–373.0]	373.0 [271.0–516.5]	221.0 [166.0–283.0]
Direct bilirubin, median [IQR], µmol/L	6.0 [4.0–8.0]	6.0 [4.5–8.5]	9.0 [1.0–9.0]
Radiological findings of lung damage, No. (%)	104/121 (86.0)	49/50 (98.0)	55/71 (77.5)
NEWS2, median [IQR]	4 [1–6]	6 [4–8]	2 [0–4]
COVID-GRAM, median [IQR]	128.5 [108.6–148.3]	137.0 [128.5–155.8]	117.2 [88.7–134.1]

*NEWS* national early warning score; *SD* standard deviation; *IQR* interquartile range

Among the total cohort, 54.5% referred dyspnoea, and it was more observed in the critical group (72.0% versus 42.3% of non-critical patients, *p* = 0.001).

We observed an increased neutrophil–lymphocyte ratio in total population (median 4.4, IQR 2.7–8.2) and it is significantly higher in critical patients (median 7.6, IQR 4.1 versus median 3.8, IQR 2.1–5.3, *p* < 0.0001).

Lactate dehydrogenase was increased in the total population (median 265 U/L, IQR 190–373 U/L) and higher in patients with severe COVID-19 compared to non-severe disease (median 373.0 U/L, IQR 271.0–516.5 U/L versus 221.0 U/L, IQR 166.0–283.0 U/L, *p* < 0.0001).

NEWS and COVID-GRAM data had not a Gaussian distribution. For both the tools, Mann–Whitney *U* test confirmed the differences of scores between patients with severe and not severe COVID-19 (*p* < 0.0001).

**Fig. 2:**
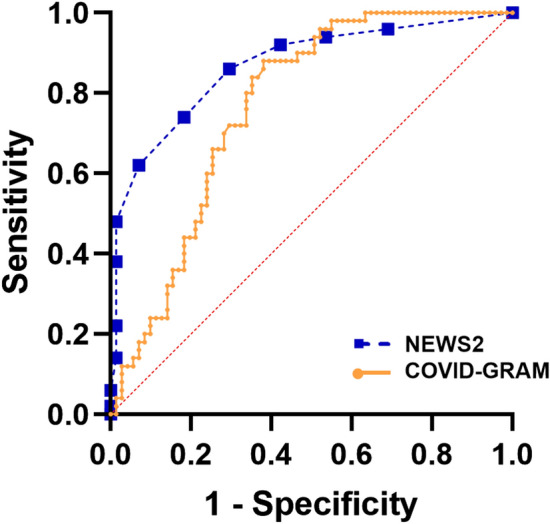
Receiver-operating characteristic (ROC) curves for prediction of critical COVID-19 illness: ROC curve for critical COVID-19 illness using NEWS2 (■ dashed line) and COVID-GRAM (• continuous line) of COVID-19 patients at hospital admission. NEWS2 showed an area under the ROC curve (AUROC) curve of 0.87 (standard error, SE 0.03; 95% CI 0.80–0.93; *p* < 0.0001). The COVID-GRAM AUROC curve measured 0.77 (SE 0.04; 95% CI 0.68–0.85; *p* < 0.0001)

The ROC curves of NEWS2 and the new COVID-GRAM for predicting critical illness are depicted in Fig. 2. The NEWS2 AUROC curve measured 0.87 (standard error, SE 0.03; 95% CI 0.80–0.93; *p* < 0.0001). The new specific COVID-GRAM AUROC curve measured 0.77 (SE 0.04; 95% CI 0.68–0.85; *p* < 0.0001). Hanley–McNeil test showed that NEWS2, better predicted severe COVID-19 (Z = 2.03).

According to the Youden index (*J*), the optimal thresholds were 4 and 123.2 for NEWS2 and COVID-19 score, respectively. As shown in Table 2, at the selected thresholds, sensitivity was 86 and 88 for NEWS2 and Liang’s COVID-GRAM, respectively. Specificity was higher for NEWS2 (70.4%) compared to COVID-GRAM (62%). NEWS2 at 4 had a positive likelihood ratio of 2.9 while for COVID-GRAM of 2.3. Similarly, positive predictive value was higher for NEWS2 (67.2%) respect to COVID-GRAM (62.9%). The negative likelihood ratios were 0.2 for both the scores. Similar negative predictive value was found for the scores (87.7% and 88.2% for NEWS2 and COVID-GRAM, respectively). Accuracy was 76.9 and 72.7 for NEWS2 and Liang’s score, respectively.

**Table 2:** Prognostic accuracy of NEWS2 and Liang’s COVID-19 score for severe COVID-19 using the optimal threshold values individuated by Youden index (*N* = 121)

(95% CI)	NEWS2 ≥ 4	COVID-GRAM (Liang) ≥ 123.2
Sensitivity, %	86.0 (73.3–94.2)	88.0 (76.2–94.4)
Specificity, %	70.4 (58.4–80.7)	62.0 (50.3–72.4)
Positive likelihood ratio	2.9 (2.0–4.2)	2.3 (1.7–3.2)
Negative likelihood ratio	0.2 (0.1–0.4)	0.2 (0.1–0.4)
Positive predictive value, %	67.2 (58.4–74.9)	62.9 (55.1–70.0)
Negative predictive value, %	87.7 (78.0–93.5)	88.2 (77.6–94.2)
Accuracy, %	76.9 (68.3–84.0)	72.7 (63.9–80.4)

*NEWS* national early warning score; *COVID-19* Coronavirus Disease 19; *CI* confidence interval

Data of sensitivity, specificity, positive and negative likelihood ratio, positive and negative predictive values and accuracy of optimal clinical thresholds are shown in Supplementary Table 1.

## Discussion

Liang et al. generated a specific COVID-19 score based on characteristics of patients of a nationwide Chinese cohort. Before this study, other authors underlined the importance of an early severity of illness assessment. For example, Bradley et al. [9] in a cohort of 830 patients, compared rapid scores as CURB-65, NEWS2 and qSOFA and observed that NEWS2 ≥ 5 had a negative predictive value of 98.0% for early mortality. However, they underlined the necessity of a COVID-19 score that focus on respiratory failure rather than circulatory collapse.

In a previous study, we demonstrated that, in COVID-19 patients, NEWS2 is useful to quickly identify patients at risk of rapid deterioration and to predict ICU admission. Furthermore, according to this study, the use of the score could be implemented basing on two different thresholds: 5 and 7 [2]. Furthermore, another study performed analysing an UK cohort of 296 patients supported the use of NEWS2 to identify deterioration of hospitalised COVID-19 patients [10].

In this study, through the Youden Index, we individuated for simplicity a threshold with a good balance of sensitivity and specificity. It was calculated to compare NEWS2 and COVID-GRAM, but it does not represent the best clinical threshold. In clinical practice, two thresholds (5–7 and 56.6–138.4 for NEWS2 and COVID-GRAM, respectively) could better fit the necessity of rapid individuation of patients at risk of deterioration and exclusion of clinically stable patients. Indeed, for both scores, the lower threshold could better individuate patients with low risk of critical progression with a sensitivity of 100% and 86.0% for COVID-GRAM and NEWS2, respectively. At the same time, the higher threshold better predicted the risk of critical course (specificity of 78.9% and 98.6% for COVID-GRAM and NEWS2, respectively) (Supplementary Table 1).

However, 79 patients had a COVID-GRAM score between the two thresholds, with a medium risk of progression; among these, 31.6% had a severe COVID-19. Concerning NEWS2, only 25 patients had a score between the two thresholds and, among these, 52.0% developed severe COVID-19 (data not shown).

We observed that NEWS2 applied for identification of patients with a high risk of deterioration in different clinical setting [5] could identify patients at risk of severe COVID-19 better than the COVID specific Liang’s Score. Of note, although COVID-19 is a disease involving mainly the lungs, among the ten variables required by COVID-GRAM, respiratory rate, oxygen saturation, and supplemental oxygen are not considered differently from NEWS2.

Multiple factors need to be discussed. First, the two cohorts of patients are different: the mean age of our patients is higher than in Liang ones (49 versus 65 years). Interestingly, in our cohort, age is similar in critical and non-critical patients. In Liang’s cohort, the mean age was higher in critical than in non-critical patients. Moreover, 51.2% of our patients have at least one coexisting condition, as opposed to 25.1% in Liang’s cohort; a higher percentage of our patients, comparing to the Chinese cohort, developed a severe disease (41.3% versus 8.2%) and had abnormal chest radiological findings (86.0% versus 71.0%).

Unfortunately, with published data, we are not able to evaluate NEWS2 in Liang’s cohort. The main strengths of this study are the homogeneity of the cohort and the setting as a double-centre study. The NEWS2 is a more practical tool that have been largely validated in other clinical setting for identification of patients with a high risk of deterioration and shows a good applicability to identify the critical COVID-19. The main limitations of our study are the retrospective study design and the small number of patients.

In conclusion, both scores provide useful information to manage patients with COVID-19, but NEWS2 seemed to be simpler and to better predict than COVID-GRAM the progression to critical COVID-19.

## Supplementary Information

Below is the link to the electronic supplementary material.

Supplementary file1 (DOCX 13 KB)

## Data Availability

Available on reasonable request.
