# Supplementary material for: National Early Warning Score 2 (NEWS2) better predicts critical Coronavirus Disease 2019 (COVID-19) illness than COVID-GRAM, a multi-centre study
Source: Infection. 2021 May 10;49(5):1033–8. doi: 10.1007/s15010-021-01620-x (PMC8108728; doi:10.1007/s15010-021-01620-x)
Supplement: Supplementary file 1 — Supplementary file1 (DOCX 13 KB) [file 15010_2021_1620_MOESM1_ESM.docx]

**Supplementary Table 1:** Prognostic accuracy of NEWS2 and Liang’s COVID-19 score for severe COVID-19 using the optimal clinical threshold values (N=121).

|  | **NEWS2 ≥5** | **NEWS2 ≥7** | **COVID-GRAM ≥56.6** | **COVID-GRAM ≥138.4** |
| --- | --- | --- | --- | --- |
| Sensitivity, % (95% CI) | 74.0 (59.7-85.4) | 48.0 (33.7-42.6) | 100.0 (92.3-100.0) | 50.0 (35.5-64.5) |
| Specificity, % (95% CI) | 81.7 (70.7-89.9) | 98.6 (92.4-100.0) | 1.4 (0.0-7.6) | 78.9 (67.6-87.7) |
| Positive likelihood ratio (95% CI) | 4.0 (2.4-6.8) | 34.1 (4.8-243.8) | 1.0 (0.9-1.0) | 2.4 (1.4-4.0) |
| Negative likelihood ratio (95% CI) | 0.3 (0.2-0.5) | 0.5 (0.4-0.7) | 0 | 0.6 (0.5-0.9) |
| Positive predictive value, % (95% CI) | 41.3 (32.5-50.6) | 96.0 (77.0-99.4) | 41.7 (41.0-42.3) | 62.5 (49.6-73.9) |
| Negative predictive value, % (95% CI) | 74.0 (62.9-82.7) | 72.9 (67.3-77.9) | 100.0 | 69.1 (62.4-75.2) |
| Accuracy, % (95% CI) | 78.5 (70.5-85.5) | 77.7 (69.2-84.8) | 42.2 (33.2-51.5) | 66.9 (57.8-75.2) |

Abbreviations: NEWS, National Early Warning Score; COVID-19 Coronavirus Disease-19; CI, confidence interval.
